# Impact of hyperkalaemia on renin–angiotensin–aldosterone (RAAS) inhibitor reduction or withdrawal following hospitalisation

**DOI:** 10.1007/s10238-024-01531-9

**Published:** 2024-12-21

**Authors:** Hugh Logan Ellis, Mohammad Al-Agil, Philip A. Kelly, James Teo, Claire Sharpe, Martin B. Whyte

**Affiliations:** 1 Department of Medicine, King’s College Hospital NHS Foundation Trust, London, UK; 2 Department of Basic and Clinical Neuroscience, School of Neuroscience, King’s College London, London, UK; 3 Department of Renal Medicine, King’s College Hospital NHS Foundation Trust, London, UK; 4 Education Centre, School of Medicine, University of Nottingham, Nottingham, UK; 5 Department Clinical and Exp Medicine, Faculty of Health and Medical Sciences, University of Surrey, Leggett Building, Daphne Jackson Road, Guildford, UK

**Keywords:** Hyperkalaemia, Renin–angiotensin system, ACE inhibitors, Mortality

## Abstract

**Background:**

Inhibitors of the renin–angiotensin–aldosterone system (RAAS), such as ACE inhibitors (ACEi), angiotensin-II receptor blockers and mineralocorticoid receptor antagonists, reduce morbidity and mortality in hypertension, congestive heart failure and chronic kidney disease. However, their use can lead to hyperkalaemia. We examined the proportions of RAAS inhibitor (RAASi) reduction or withdrawal, across GFR strata, following hospitalisation and the effect on patient mortality.

**Methods:**

This was a retrospective cohort study of adult patients hospitalised from 1 January2017 to 31 December2020. Biochemistry data, clinical notes and medicines use were extracted using the CogStack platform, from electronic health records. Patients were identified by creatinine measurement during hospitalisation. Hyperkalaemia was defined as potassium > 5.0 mmol/L, with severity categorisation. RAASi discontinuation defined as ≥ 48 h without administration. Mortality risk associated with RAASi cessation was assessed using Cox proportional hazards models.

**Results:**

Among 129,172 patients with potassium measurements, 49,011 were hospitalised. Hyperkalaemia prevalence was 8.57% in the emergency department and 16.79% among hospitalised patients. Higher hyperkalaemia levels correlated with increased CKD and heart failure. RAASi use was more common in hyperkalaemic patients, with higher discontinuation rates during hospitalisation (36% with potassium 5–5.5 mmol/L; 61% with potassium > 6.5 mmol/L). By discharge, 32% of patients had RAASi stopped, and 2% doses reduced. Discontinuation of RAASi was associated with 37% worse survival probability.

**Conclusion:**

RAASi cessation was greater with hyperkalaemia and associated with increased mortality in hospitalised patients. Reinstitution of RAASi after hospital discharge, or alternative management of hyperkalaemia if maintained on RAASi therapy, may improve clinical outcomes.

## Background

Drugs that inhibit the renin–angiotensin–aldosterone system (RAAS inhibitors), such as angiotensin-converting enzyme inhibitors (ACEi), angiotensin-II receptor blockers (ARB) and mineralocorticoid receptor antagonists (MRA), reduce the morbidity and mortality of hypertension [1], congestive heart failure (CHF) with reduced ejection fraction [2] and reduce proteinuria and slow the decline of estimated glomerular filtration rate (eGFR) in chronic kidney disease (CKD) [3]. International guidelines recommend that RAAS inhibitor therapy is titrated to the moderate-to-high doses that have been used in clinical trials to derive the maximum clinical benefit [1, 4, 5]. However, inhibition of RAAS can lead to hyperkalaemia [6]. Potassium > 5 mmol/L has been seen in ~ 20% of patients on ACEi and 31% on ARBs, whereas severe hyperkalaemia (6 mmol/L or higher) has been observed in 0.8% of ACEi and 2.8% of ARB users [7]. ACEi and ARB therapy has been considered a contributing cause in 10% to 38% of hospitalised hyperkalaemia cases [8–11]. Hyperkalaemia is a potentially serious condition that can result in life-threatening cardiac arrhythmias and is associated with an increased mortality risk [12, 13]. The presence of hyperkalaemia may therefore lead to down-titration or discontinuation of RAAS inhibitors (RAASi) [14, 15]; this may lead to adverse cardiovascular morbidity and mortality.

We examined the effect of withdrawal of RAASi following hospitalisation. We hypothesised that RAASi withdrawal would be associated with worse mortality.

## Methods

This retrospective cohort study included all adults attending King’s College Hospital (KCH) with an unscheduled, acute hospitalisation and who had a biochemical measurement of kidney function (urea and electrolyte blood test, U&E). KCH is a 950-bed, acute-care tertiary hospital. The local catchment area has a population of 1.2 million. CogStack is an information retrieval, extraction and natural language processing platform. CogStack can extract information from unstructured text within medical records, including patient notes, discharge summaries and diagnostic reports. We used the CogStack ecosystem to identify all hospital admissions from 1st January 2017 to 31st December 2020 and to access structured fields in the patient-level electronic health record (EHR).

All inpatients with U&E blood measurement were identified. From these, we then identified the numbers using ACEi, ARB, MRA as well as other drugs, including beta-blockers, non-steroidal anti-inflammatory drugs (NSAIDs) and thiazide- and loop-diuretics. Pre-hospital drugs and doses were identified from the pharmacy reconciliation which is entered into the EHR and retrieved using CogStack. From this, we were able to determine any dose changes or drug cessation/initiation, in hospital.

The study period chosen was based on the availability of complete data across the hospital system. We used data captured through routine care in a single EHR instance (Sunrise Clinical Manager, Allscripts). Inclusion criteria were hospitalised patients at King’s College Hospital between 1st Jan 2017 and Dec 31st 2020. Patients < 18 years of age were excluded.

### Definitions

Hyperkalaemia was defined as > 5.0 mmol/L; however, we further report outcomes according to the severity of hyperkalaemia episode, as serum potassium > 5.5 mmol/L, > 6.0 mmol/l and > 6.5 mmol/L. The index date and time was defined as the first hyperkalaemic potassium measurement (either up to 24 h prior to hospitalisation, or during hospitalisation). RAASi discontinuation was defined as ≥ 48 h without RAASi administration. The 48-h window was based on timing of RAASi discontinuation (± restart) in studies of patients undergoing elective procedures [16] and allows for involuntary non-administration of a dose. Dose reduction was defined as the presence of a dose lower than the most recent RAASi prescription, prior to the index date. CKD severity was based on the estimated glomerular filtration rate. Acute kidney injury was determined from the free text alert that automatically accompanies a U&E result that is acutely out of range. The text was identified using the Medical Concept Annotation Tool ‘MedCAT’.

The frailty index-laboratory (Fi-Lab) was used as an electronic index of frailty [17]; it was created at admission from 26 common laboratory tests. Each test is categorised as normal or abnormal based on the local reference intervals. The Fi-lab is a score of the proportion of abnormal results from the total number of tests ordered. It has been shown to relate well to clinical frailty, in relation to short-term outcomes (i.e. length of stay, a higher level of care upon discharge and in-hospital mortality) and long-term outcomes [17]. Admissions on the same day as discharge or transfers between departments were considered as a single hospitalisation.

### Statistical methods

Descriptive statistics of the hospitalised cohort were made using mean and 95% confidence interval; median and inter-quartile range for continuous data and counts and proportions (%) for categorical data. Independent sample tests of categorical variables against outcomes were tested with Chi-square tests of significance for categorical variables. Statistical tests were conducted using the maximum potassium recorded.

A Cox proportional hazards models were constructed to evaluate the independent effect of RAASi cessation on mortality, adjusting for age, sex, Fi-lab, first creatinine, maximum potassium, presence of acute kidney injury, presence of diabetes mellitus and palliative care involvement. Analyses were performed using R statistical software, version 3.6.1.

### Laboratory methods

Potassium values may be obtained and/or actioned based-upon ‘point-of-care’ venous blood gas samples. KCH does not use capillary samples for biological measures, other than for glucose or ketone testing. For this study, such point-of-care (‘blood gas’) results were utilised, as these are often the trigger for clinical intervention. Blood gas data were also accessed through CogStack system. These were analysed and found to be within clinically meaningful range of the “true” laboratory result > 99% of the time, and the overwhelming majority had a laboratory sample taken simultaneously [18]. It was considered that reliance solely on laboratory values could be biased as such samples may derive values after clinical intervention.

### Ethical approval

The King’s College Hospital Research and Innovation Department advised that this was a service evaluation, rather than research. It was felt that it was not research. We confirmed this opinion using the HRA ‘Is this research?’ decision tool (http://www.hra-decisiontools.org.uk/research/). Local approval for use of CogStack EPR searched was sought from the King's Electronic Patient Record Interface (KERRI) committee (project ID 20210405A), and approval was received on 7th May 2021.

## Results

In total, there were 129,172 patients who had a blood test (involving a potassium measure) taken over the study period, which led to 49,011 admissions. A summary table of the patient characteristics, for all the visits where there was a potassium measurement, is shown (Table 1).

**Table 1:** Characteristics of study cohort

Characteristic	Value
Total number of potassium tests	129,172
Sex (N, %)	
Male	60,953 (47.2)
Female	68,201 (52.8)
Both	< 10
Indeterminate	< 10
Missing	< 10
Age years; mean (SD)	50 (24)
Total admissions (n)	49, 011
Admissions per patient; median (IQR)	1 (1–3)
Length of stay days; median (IQR)	0.4 (0.2–2.7)
Time to readmission; days; median (IQR)	219 (43–516)
Died during follow-up; n (%)	
Yes	8355 (6.5)
No	120,817 (93.5)
Of those who died, died in-hospital? N (%)	
Yes	2,322 (27.8)
No	6,033 (72.2)

Of all the patients who had blood tests within the emergency department, the prevalence of hyperkalaemia was 85.7 per 1000. Of those patients who were admitted to hospital (defined as length of stay > 24 h), the prevalence of hyperkalaemia was 167.9 per 1000 patients. The proportion of patients with hyperkalaemia and eGFR < 30 ml/min/m^2^ and eGFR < 60 ml/min/m^2^ was then further evaluated by the presence or absence of acute kidney injury (AKI), and data were further categorised by serum creatinine threshold (Table 2). Thirty-five per cent of those whose maximum in-hospital potassium was > 5.0 to 5.5 mmol/L and eGFR < 30 ml/min/m^2^ had AKI. This rose to 63% of those with maximum in-hospital potassium up to 6.5 mmol/L and eGFR < 30 ml/min/m^2^.

**Table 2:** Proportion of patients with hyperkalaemia, by eGFR, creatinine and acute kidney injury (AKI) status

Maximum hyperkalaemia	AKI	N	Proportion eGFR < 30 ml/min/m^2^	Proportion eGFR < 60 ml/min/m^2^	Proportion creatinine > 150 µmol/L
(5,5.5]	FALSE	5,178	0.11 (0.10, 0.12)	0.40 (0.39, 0.41)	0.15 (0.14, 0.16)
(5,5.5]	TRUE	1,381	0.35 (0.32, 0.37)	0.83 (0.81, 0.85)	0.47 (0.45, 0.50)
(5.5,6]	FALSE	1341	0.28 (0.26, 0.31)	0.58 (0.56, 0.61)	0.34 (0.32, 0.37)
(5.5,6]	TRUE	812	0.44 (0.40, 0.47)	0.87 (0.84, 0.89)	0.55 (0.52, 0.59)
(6,6.5]	FALSE	436	0.47 (0.42, 0.52)	0.74 (0.70, 0.78)	0.52 (0.47, 0.57)
(6,6.5]	TRUE	401	0.50 (0.45, 0.55)	0.90 (0.87, 0.93)	0.63 (0.58, 0.68)
(6.5,Inf]	FALSE	349	0.58 (0.52, 0.63)	0.75 (0.70, 0.79)	0.61 (0.56, 0.66)
(6.5,Inf]	TRUE	353	0.65 (0.59, 0.70)	0.90 (0.86, 0.93)	0.73 (0.68, 0.77)

‘TRUE’ signifies that AKI was present. ‘FALSE’ that AKI was absent

Data are mean (95% CI)

For the degree of hyperkalaemia, square brackets signify the number is included

We determined the burden of comorbidities associated with hyperkalaemia (Table 3). This showed escalating proportions of CKD and heart failure in higher hyperkalaemic strata. Patients with diabetes comprised 20% of those with potassium > 5.0 – 5.5 mmol/L and ≥ 30% in those with potassium > 5.5 mmol/L.

**Table 3:** The proportion of comorbidities per strata of hyperkalaemia

Hyperkalaemia category(mmol/L)	n	ESRF	ESRF on dialysis	Diabetes	CKD	Heart failure
*Percentage of patients within each strata per comorbidity (95% confidence interval)*						
(5.0 to 5.5]	7148	5 (4, 5)	3 (3, 3)	20 (19, 21)	21 (20, 22)	29 (28, 30)
(5.5 to 6.0]	2313	12 (11, 14)	9 (8, 10)	30 (28, 32)	35 (34, 37)	40 (38, 42)
(6.0 to 6.5]	876	21 (19, 24)	17 (15, 20)	34 (31, 37)	49 (46, 52)	47 (44, 50)
(6.5 to Inf]	733	32 (28, 35)	24 (21, 28)	32 (29, 36)	54 (50, 57)	48 (45, 52)

For the degree of hyperkalaemia, square brackets signify the number is included

Numbers in parentheses are 95% CI for the mean

*CKD* chronic kidney disease, *ESRF* end-stage renal failure

The proportion of each stratum of eGFR using ACEi, ARB, beta-blockers or MRA at the time of admission were further categorised by the presence or absence of hyperkalaemia. For each eGFR stratum, the proportion of ACEi use in those with hyperkalaemia was greater than in those with normokalaemia (Table 4). Correspondingly, for any eGFR stratum, the presence of hyperkalaemia was associated with a greater proportion of ACEi discontinuation during the hospitalisation. Similar trends were seen with ARB and MRA but not with beta-blockers. Only with eGFR < 15 ml/min/m^2^ was there little difference in those continuing *vs* discontinuing ACEi.

**Table 4:** The proportion of patients prescribed each class of medication on admission, and of those the proportion whose medication was ceased, and not restarted by discharge

eGFR (ml/min/m^2^) at time of maximum potassium	Maximum potassium during hospitalisation	n	% using ACEi on admission	% who ceased ACEi	% using ARB on admission	% who ceased ARB	% using MRA on admission	% who ceased MRA	% using beta-blocker on admission	% who ceased beta-blocker
< 15	≤ 5 mmol/L	475	21	44	10	54	4	40	47	18
	hyperkalaemia	954	25	46	15	42	3	50	47	19
15–29	≤ 5 mmol/L	1103	22	45	15	36	5	38	36	17
	hyperkalaemia	930	25	57	15	57	8	58	41	20
30–44	≤ 5 mmol/L	2488	26	33	16	29	5	34	32	17
	hyperkalaemia	1220	30	46	14	42	8	44	38	19
45–59	≤ 5 mmol/L	4438	23	24	12	22	4	20	26	15
	hyperkalaemia	1110	29	31	12	35	7	43	30	18
≥ 60	≤ 5 mmol/L	22,081	13	21	5	23	2	20	13	16
	Hyperkalaemia	2582	18	27	6	28	5	28	18	15

Grouping ACEi, ARB and MRA together as a single entity (‘RAAS inhibitor’) and including all patients (normo- and hyperkalaemic), it was seen that by the time of hospital discharge 32% had a RAAS inhibitor stopped and 2% reduced, compared to 17% started on a RAAS inhibitor and 5% increased. Whether RAAS drugs were reduced, or stopped, for any given potassium stratum, is shown in Appendices 1–3. Of those with potassium > 6.5 mmol/L, nearly two-thirds achieved normalisation of potassium prior to hospital discharge, whereas that figure was ~ 40 to 50% for lower strata of hyperkalaemia. This may reflect greater proportions of RAAS drugs being stopped in the higher potassium strata (Appendix 2 & 3).

### The effect of cessation of RAAS

**Fig. 1:**
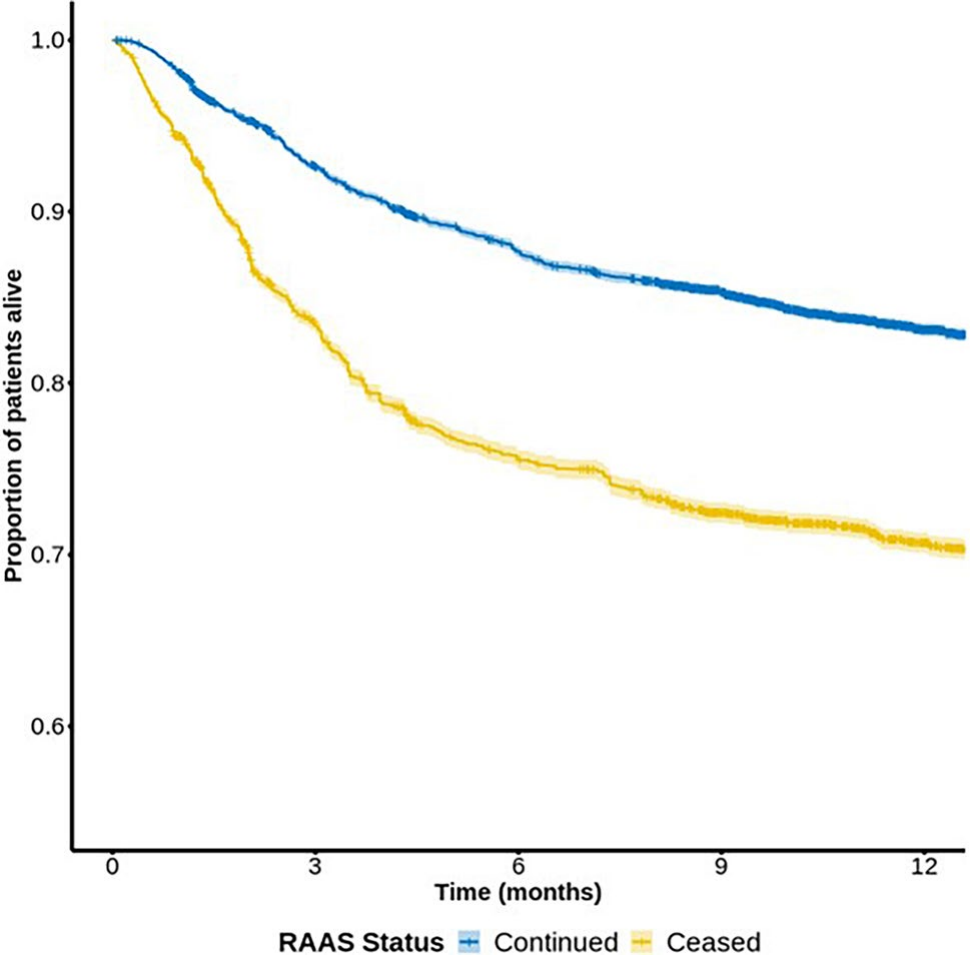
The risk of death in patients who had RAASi ceased at hospital discharge

Crude data, showing the unadjusted association of RAAS discontinuation and survival, is shown in Fig. 1. There was a significantly reduced chance of survival in those who had RAASi stopped by the date of the last analysis (5th April 2021). In the adjusted model (Table 5), the relationship of RAASi cessation and mortality persisted. This remained the case in a sensitivity analysis with Fi-Lab (an index of frailty) removed from the model (data not shown).

**Table 5:** Multivariable Cox model evaluating the effect of RAASi cessation on risk of death

	Coefficient (95% confidence interval)	P value
RAASi stopped	1.370 (1.254–1.497)	< 0.001
Fi-Lab	1.023 (1.019–1.027)	< 0.001
First creatinine (µmol/L)	0.999 (0.999–0.999)	< 0.001
Maximum potassium (mmol/L)	1.109 (1.047–1.175)	< 0.001
AKI (true)	1.041 (0.947–1.144)	0.4075
Age (years)	1.016 (1.013–1.019)	< 0.001
Diabetes mellitus	0.811 (0.744–0.883)	< 0.001
Male	0.636 (0.585–0.691)	< 0.001
Palliative care	3.559 (3.180–3.984)	< 0.001

*AKI* acute kidney injury, *RAASi* renin–angiotensin–aldosterone system inhibitors

## Discussion

This observational study has shown that cessation of RAASi, in the context of hyperkalaemia, is associated with worse mortality. It is generally advised that if the serum potassium increases above 5.5 mmol/L, the ACEi/ARB should be discontinued [19]. Managing hyperkalaemia in patients with cardiorenal diseases therefore poses a challenge for clinicians. Electing to not use RAASi treatment in this patient population may lead to poor health outcomes and increased healthcare costs [20].

Observational studies in community settings have shown that more than half of the individuals initiating ACE-I or ARB therapy discontinued within 5 years after the initial prescription, with discontinuation increasingly common with more advanced CKD stage [21, 22]. This is worrying in view of a meta-analysis showing that ACE-/ARB reduces the risk of kidney failure and cardiovascular events in CKD [23]. Since that meta-analysis, observational data from the Veterans Affairs healthcare system in USA suggest that in CKD Stages 3 and 4, ACEI/ARB discontinuation was independently associated with an increased risk of subsequent death and end-stage kidney disease (ESKD) [24]. Two further studies have shown that ACEi discontinuation for Stage 4 CKD was also associated with worse major adverse cardiovascular events (MACE)[25, 26] and progression to ESKD in a population with diabetes[26], but not in a general community cohort[25]. The multi-centre randomised controlled trial of Angiotensin-Converting Enzyme Inhibitor/Angiotensin Receptor Blocker Withdrawal in Advanced Renal Disease (STOP-ACEi) found that the discontinuation of RAAS inhibitors was not associated with a significant difference in the long-term rate of decrease in the eGFR in CKD stages 4 to 5 [27]. There is equipoise about the relationship between ACEI/ARB and AKI [28]. However, in terms of potassium as a driver for cessation Leon et al. [29] found that patients who stopped taking the drugs following an episode of hyperkalaemia had a ~ 1.4 -fold increased risk of all-cause and ~ 1.3-fold risk of cardiovascular mortality, in adjusted analyses compared with patients who continued taking the medications. Our data tell a similar story with a 1.66 adjusted hazard ratio for death. These data will help guide practice.

It is known that in-hospital hyperkalaemia is associated with greater mortality [30]. Patients with a potassium of 5.1 to 5.5 mmol/L have twice the risk of dying in hospital than those with levels between 3.5 and 5.0 mmol/L [31]. Our data show 14-day mortality in the > 6 mmol/L stratum was near double that of the 5—6 mmol/L stratum. Furthermore, hyperkalaemia remained an independent predictor after adjustment for confounding variables.

Unexpectedly, we found no discernible trends in the proportion of patients prescribed ACEI, ARB etc. across the potassium strata. However, our data do conform to those published previously that those with hyperkalaemia are much more likely to have CKD, diabetes and heart failure [20, 32]. In our series, the relative proportion with diabetes showed stepwise increase with escalating level of potassium [6]. Also in our series, the proportion of ACEi having a dose reduction or cessation was approximately one-third whose potassium was 5.0–5.5 mmol/L, increasing to two-thirds in those whose potassium was over 6.5 mmol/L.

### Limitations

We did not have access to the local care record (for primary care records) which meant that we could not precisely determine the proportion having RAASi drugs reinstated by 1-month, after hospital discharge. We estimated that near three-quarters of those who were discharged but were later readmitted, had had RAASi reinstated prior to the readmission. However, these data would have intrinsic bias and may not be a representative sample of all data.

We were not able to access the software platform that housed the clinical observations. Therefore, the National Early Warning Score (NEWS) could not be determined. This meant that ‘illness severity’ could not be included in the regression models of death and readmission from hyperkalaemia.

We have defined hyperkalaemia as a serum potassium concentration higher than 5.0 mmol/L. Severe hyperkalaemia has been defined as a serum potassium concentration higher than 6.5 mmol/L [33]. In practice, hyperkalaemia is a continuum. An indicator of hyperkalaemia may also be the presence of ECG abnormalities [34], but we did not have the ability to ‘read’ ECGs with available software.

Serum potassium will increase as serum pH decreases because potassium shifts from the cellular to the vascular space—to what degree hyperkalaemia is a pathophysiological bystander for co-incident acidaemia is uncertain and further work is needed to establish this. It is noteworthy that a third of individuals with maximum potassium > 6.5 mmol/L had an eGFR of > 30 ml/min/m^2^. Whether hyperkalaemia directly led to admissions, for example, through muscle weakness, cardiac problems, resulting falls, is uncertain. This is especially true with comorbidity [20]—in our cohort, heart failure, diabetes and kidney failure were all over-represented in those with hyperkalaemia.

For precision, we only included laboratory measures of potassium, although the accuracy of point-of-care can be within 0.1–0.5 mmol/L [35].

## Conclusion

In conclusion, the cessation of RAASi, in the context of hyperkalaemia, is associated with worse mortality. Clinicians should carefully weigh, with their patients, the risks and benefits when considering the cessation of RAASi. Our data underscore the importance of continued adherence to prescribed RAASi therapy to optimise long-term health outcomes.

## Data Availability

The code used to interrogate the data is available on request from corresponding author. The datasets generated and analysed during the current study are not publicly available as the data comprise individual medical health records. Access to the clinical data can only be made on-site at King’s College Hospital, via an application to the KERRI committee (Prof James Teo, King’s College Hospital).

## Appendix 1: Percentage of patients prescribed each medication on admission with dose reduced (but not stopped) by discharge

**Table:**

Drug name	Hyperkalaemia			
	(5.0 to 5.5]	(5.5 to 6.0]	(6.0 to 6.5]	(6.5 to Inf]
*Total number*	4258	1573	614	524
Atenolol	2 (0, 9)	6 (1, 21)	5 (0, 28)	10 (1, 46)
Bisoprolol	9 (8, 11)	7 (5, 9)	5 (2, 9)	11 (7, 17)
Candesartan	4 (1, 8)	2 (0, 10)	0 (0, 23)	10 (2, 33)
Irbesartan	0 (0, 6)	9 (2, 25)	5 (0, 25)	6 (1, 22)
Lisinopril	4 (1, 10)	0 (0, 13)	0 (0,20)	0 (0, 34)
Losartan	4 (2, 8)	8 (3, 19)	4 (0, 24)	21 (6, 51)
Perindopril	0 (0, 14)	0 (0, 19)	0 (0, 60)	0 (0, 95)
Propranolol	6 (2, 18)	5 (0, 26)	10 (1, 46)	0 (0, 69)
Ramipril	4 (2, 5)	4 (3, 7)	3 (1, 8)	2 (0, 8)
Spironolactone	5 (2, 8)	4 (1, 11)	6 (1, 21)	0 (0, 17)

For the degree of hyperkalaemia, square brackets signify the number is included.

Numbers in parentheses are 95% CI for the mean.

## Appendix 2: Numbers and categories of pre-admission drugs stopped during admission

**Table:**

Percentage of patients who had a drug in each class ceased or reduced							
Hyperkalaemia category (mmol/L)	n	ACEi	Beta-blocker	ARB	NSAID	MRA	Insulins
(5.0 to 5.5]	4258	36 (33, 39)	25 (22, 27)	37 (32, 41)	57 (51, 63)	36 (30, 43)	33 (29, 38)
(5.5 to 6.0]	1573	48 (43, 53)	25 (22, 29)	49 (41, 57)	62 (50, 73)	61 (50, 70)	36 (30, 42)
(6.0 to 6.5]	614	54 (47, 62)	26 (21, 33)	70 (57, 80)	68 (43, 86)	69 (52, 83)	37 (29, 46)
(6.5 to Inf]	524	61 (52, 70)	31 (25, 38)	66 (53, 76)	59 (33, 81)	56 (35, 75)	37 (28, 48)

For the degree of hyperkalaemia, square brackets signify the number is included.

Numbers in parentheses are 95% CI for the mean.

ACEi: angiotensin-converting enzyme inhibitor; ARB: angiotensin receptor blocker; MRA: mineralcorticoid receptor antagonist; NSAID: non-steroidal anti-inflammatory drug.

## Appendix 3: The proportion of preadmission RAAS drugs with either dose reduction, or cessation, by hospital discharge

**Table:**

	Hyperkalaemia category			
	(5.0 to 5.5]	(5.5 to 6.0]	(6.0 to 6.5]	(6.5 to Inf]
*Number of patients*	4,258	1,573	614	524
Atenolol	34 (25, 45)	51 (34, 68)	42 (0.21, 0.66)	70 (35, 92)
Bisoprolol	15 (13, 18)	18 (15, 22)	20 (0.15, 0.27)	20 (0.14, 0.27
Bumetanide	20 (14, 27)	38 (28, 49)	55 (0.39, 0.7)	41 (21, 63)
Candesartan	37 (30, 45)	42 (29, 55)	71 (0.44, 0.89)	60 (36, 80)
Enalapril	31 (19, 46)	58 (37, 76)	67 (0.24, 0.94)	80 (30, 99)
Fludrocortisone	27 (7, 61)	0 (0, 69)	50 (0.09, 0.91)	25 (1, 78)
Furosemide	36 (32, 40)	40 (34, 46)	45 (0.36, 0.55)	47 (35, 59)
Ibuprofen	49 (39, 58)	58 (39, 75)	60 (0.17, 0.93)	67 (24, 94)
Irbesartan	36 (26, 48)	35 (20, 54)	64 (0.41, 0.82)	52 (34, 69)
Lisinopril	35 (26, 45)	61 (42, 77)	65 (0.41, 0.84)	100 (66, 100)
Losartan	31 (25, 39)	48 (36, 61)	70 (0.47, 0.86)	57 (30, 81)
Metoprolol	50 (25, 75)	40 (7, 83)	100 (5, 100)	50 (9, 91)
Perindopril	53 (35, 71)	62 (39, 81)	100 (40, 100)	100 (50, 100)
Propranolol	22 (12, 37)	40 (20, 64)	30 (8, 65)	0 (0, 69)
Ramipril	42 (39, 46)	52 (47, 58)	59 (50, 67)	66 (55, 75)
Spironolactone	32 (26, 38)	57 (46, 67)	63 (45, 78)	56 (35, 75)

Numbers in parentheses are 95% CI for the mean.

For the degree of hyperkalaemia, square brackets signify the number is included.

## Abbreviations

ACEi: ACE inhibitors
AKI: Acute kidney injury
ARB: Angiotensin-II receptor blockers
CHF: Congestive heart failure
CKD: Chronic kidney disease
eGFR: Estimated glomerular filtration rate
ESKD: End-stage kidney disease
Fi-Lab: Frailty index-laboratory
KCH: King’s College Hospital
KERRI: King's Electronic Patient Record Interface
MACE: Major adverse cardiovascular events
MedCAT: Medical Concept Annotation Tool
MRA: Mineralocorticoid receptor antagonists
NSAIDs: Non-steroidal anti-inflammatory drugs
RAAS: Renin–angiotensin–aldosterone system
RAASi: RAAS inhibitor
